# Comparison of adverse renal events between ranibizumab and aflibercept in patients with diabetic macular oedema: A global network study

**DOI:** 10.1038/s41433-026-04459-4

**Published:** 2026-04-22

**Authors:** Wan-Ju Annabelle Lee, Daniel Hsiang-Te Tsai, Michael Chun-Yuan Cheng, Yu-Shiuan Lin, Chia-Yi Lee, Shih-Chieh Shao, Edward Chia-Cheng Lai

**Affiliations:** 1 Department of Ophthalmology, Chi Mei Medical Center, Tainan, Taiwan; 2 School of Pharmacy, Institute of Clinical Pharmacy and Pharmaceutical Sciences, College of Medicine, National Cheng Kung University, Tainan, Taiwan; 3 Population Health Data Center, National Cheng Kung University, Tainan, Taiwan; 4 Department of Pharmacy, Keelung Chang Gung Memorial Hospital, Keelung, Taiwan

**Keywords:** Epidemiology, Macular degeneration

## Abstract

**Purpose:**

Patients with diabetic macular oedema (DMO) are susceptible to renal injury, raising the risks of acute kidney injury (AKI) and end-stage renal disease (ESRD) associated with anti-VEGF therapies. We aimed to compare the risks of AKI and ESRD between aflibercept and ranibizumab in DMO patients, focusing on AKI and ESRD in conjunction with all-cause mortality.

**Methods:**

A target trial emulation using the TriNetX Global Collaborative Network included adult patients with DMO who initiated treatment with aflibercept or ranibizumab in routine clinical practice. Ranibizumab exposure reflected real-world use and included both approved dosages. Patients with pre-existing ESRD or chronic kidney disease stage 5 were excluded. Propensity score matching was applied to balance demographics, comorbidities, medication use, and laboratory parameters. Time-to-event analyses were performed for AKI, ESRD, and all-cause mortality. Sensitivity analyses included application of a minimum 7-day latency window after treatment initiation to address potential temporality bias.

**Results:**

After matching, 3411 patients were included in each treatment group (mean age, 64.0 ± 12.4 years; 51.5% male). During follow-up, aflibercept was not associated with an increased risk of AKI (hazard ratio [HR]: 0.97, 95% confidence interval [CI]: 0.89-1.06), ESRD (HR: 0.95, 95% CI: 0.83–1.09), or all-cause mortality (HR: 0.99, 95% CI: 0.89–1.10), when compared with ranibizumab. Results were consistent across subgroup and sensitivity analyses, including analyses incorporating a 7-day latency window with an HR of 1.08 (95% CI: 0.94–1.24) for AKI, and 0.89 (95% CI: 0.76–1.04) for ESRD.

**Conclusions:**

In this multinational, real-world study, aflibercept and ranibizumab demonstrated comparable risks of AKI, ESRD and mortality among patients with DMO after adjustment for measured confounders. Given the observational design, real-world dose heterogeneity, and limitations in detailed treatment-timing data, these findings should be interpreted with caution.

## Introduction

Diabetic macular oedema (DMO), a significant complication of diabetic retinopathy, arises when diabetes begins to affect small blood vessels of the retina. Oedematous fluid accumulates in the macula, which is the central area of the retina that allows for sharp, central vision [1]. Traditionally, the management of DMO has involved photocoagulation by laser and the injection of corticosteroids, but more recent approaches have aimed to reduce oedema, improve visual acuity, and prevent any further deterioration of vision by restraining the activity of vascular endothelial growth factor (VEGF). This is achieved using anti-VEGF agents, including aflibercept, ranibizumab and bevacizumab, which show superior efficacy compared to traditional methods, with an advantageous safety profile. Significant gains in visual acuity and decreases in macular thickness have been documented in clinical trials, and as a result, anti-VEGF agents have been widely adopted as first-line therapy for DMO.

However, acute kidney injury (AKI) and proteinuria have been reported in several case reports and studies of patients undergoing treatment for various retinal conditions using anti-VEGF agents [2–6]. A plausible physiological mechanism for such adverse renal events may be found in the disruption of normal renal vascular homeostasis as a result of disease progression or systemic VEGF inhibition [7, 8]. Intravitreally administered anti-VEGF therapy could potentially affect endothelial cells and podocytes to a degree that leads to renal injury following treatment. As a result, doctors may be unwilling to prescribe anti-VEGF agents, or DMO patients may refuse the treatment, impairing the management of their condition [9–11]. Hence, a better understanding of adverse renal events associated with the use of anti-VEGF agents for retinal conditions is needed. Specifically, our study aimed to compare the risks of adverse renal events associated with aflibercept and ranibizumab, including AKI and end-stage renal disease (ESRD) in patients with DMO. Given that previous randomized controlled trials in western countries found no difference between these two anti-VEGF agents [12], we aimed to enhance causal inference in our study by using a target trial emulation and data drawn from a global, federated health research network.

## Methods

### Data sources

The source of the data for this study was the TriNetX Global Collaborative Network, which is a globally operating, federated health research network that manages the data of approximately 129 million patients involved with 108 healthcare organisations, and which has previously provided real-world data evidence for pharmacoepidemiologic studies [13, 14]. The data for this study covered the period starting from inception of the database until May 13th, 2024, during which both aflibercept and ranibizumab were in routine clinical use across participating regions, in the form of electronic medical records (EMRs) covering diagnoses, medications, procedures, laboratory results and genomic data. This retrospective study was exempt from ethical approval. The data reviewed was a secondary analysis of existing data, did not involve intervention or interaction with human subjects, and was de-identified according to the standard defined in Section §164.514(a) of the HIPAA Privacy Rule. The process by which the data was de-identified is attested to through formal determination by a qualified expert.

### Study population

Emulating a pragmatic trial based on the TriNetX Global Collaborative Network, the specifications of which are presented in Supplementary Table 1, we included adult patients with DMO who were treated with either aflibercept or ranibizumab, excluding any with pre-existing ESRD or chronic kidney disease stage 5. The patients were identified using the International Classification of Diseases, Tenth Revision, Clinical Modification code E11.311. Depending on which drug they initiated the treatment with, we classified them as aflibercept or ranibizumab users. Ranibizumab exposure reflected routine clinical practice within the TriNetX Global Collaborative Network and therefore included both approved dosages (0.3 mg and 0.5 mg). However, dose-stratified data were not consistently available across participating healthcare organisations. Therefore, findings should be interpreted with caution.

### Outcomes and follow-up

AKI and ESRD were the primary outcomes, and all-cause mortality was the secondary outcome. Using as-started analysis as an analogue to intention-to-treat analysis, we investigated the effect on initial treatment assignment. To address the temporal relationship, our analysis adhered to a time-to-event methodology. The follow-up period started upon initiation of treatment (index date) and continued until the first occurrence of a primary outcome (AKI or ESRD), death, loss to follow-up, or administrative censoring. Events were captured throughout follow-up, starting from treatment initiation. To mitigate potential temporality and reverse-causation bias, we performed a sensitivity analysis by applying a minimum 7-day latency window after treatment initiation, during which renal events were not counted. This latency window was defined relative to the index date (first recorded anti-VEGF treatment). We included all-cause mortality as a secondary outcome to provide a broader context of systemic health status. Mortality events were identified from de-identified death records captured within the TriNetX network and represent deaths from any cause, without specification of aetiology or timing relative to anti-VEGF injection.

### Covariates

To account for any potential confounding factors, we selected relevant covariates after consulting the literature and drawing on expert opinion. The covariates included sex, age, comorbidities (including cardiovascular and liver diseases, hypertension, renal and respiratory conditions, diabetes-related conditions and ocular conditions), medications (including antihypertensive drugs, lipid-lowering agents, anti-inflammatories, diuretics, alpha blockers, antidiabetics and bevacizumab), and laboratory parameters (including systolic blood pressure, BMI, haemoglobin A1c (HbA1c) and serum creatinine). The outcomes and covariates are detailed in Supplementary Tables 2– 4.

### Statistical analysis

We mimicked randomisation between the aflibercept and ranibizumab user groups using propensity score matching to ensure a similar probability of treatment for the patients. We based the calculation of these propensity scores on the covariates listed in Table 1. We presented continuous data as the mean ± standard deviation (SD), and categorical data as numbers and frequencies. We used standardised mean differences (SMD) to evaluate divergences in baseline characteristics, whereby an SMD below 0.1 indicated a negligible difference [15]. All analyses were anchored to each patient’s treatment initiation date (index date), enabling time-to-event modelling despite the absence of exportable calendar-year distribution data. To obtain an estimate of the effects of the drugs as regards AKI or ESRD, we calculated hazard ratios (HRs) with 95% confidence intervals (CIs), based on the Cox proportional hazards model.

**Table 1:** Baseline characteristics of aflibercept and ranibizumab users.

Variables	Before PS matching			After PS matching		
	Aflibercept (*N* = 9206)	Ranibizumab (*N* = 3422)	SMD	Aflibercept (*N* = 3411)	Ranibizumab (*N* = 3411)	SMD
Age at index (years, mean ± SD)	63.7 ± 11.9	64.0 ± 12.6	0.03	63.9 ± 12.1	64.0 ± 12.6	0.01
Male sex, n (%)	4675 (50.8)	1766 (51.6)	0.02	1753 (51.4)	1761 (51.6)	0.01
Co-morbidities, n (%)						
Age-related cataract	3452 (37.5)	947 (27.7)	0.21	958 (28.1)	947 (27.8)	0.01
Other cataract	1567 (17.0)	595 (17.4)	0.01	572 (16.8)	587 (17.0)	0.01
Disorders of lipoprotein metabolism and other types of lipidaemia	4026 (43.7)	1300 (38.0)	0.12	1250 (36.6)	1295 (38.0)	0.03
Glaucoma	1873 (20.3)	551 (16.1)	0.11	542 (15.9)	549 (16.1)	0.01
Essential hypertension	5068 (55.1)	1651 (48.2)	0.14	1593 (46.7)	1647 (48.3)	0.03
Ischemic heart diseases	1599 (17.4)	534 (15.6)	0.05	500 (14.7)	532 (15.6)	0.03
Diseases of the liver	326 (3.5)	81 (2.4)	0.07	92 (2.7)	81 (2.4)	0.02
Acute myocardial infarction	247 (2.7)	75 (2.2)	0.03	66 (1.9)	75 (2.2)	0.02
Influenza and pneumonia	292 (3.2)	82 (2.4)	0.05	79 (2.3)	82 (2.4)	0.01
Acute kidney failure and chronic kidney disease	1998 (21.7)	573 (16.7)	0.13	565 (16.6)	573 (16.8)	0.01
Other disorders of the kidney and ureter	619 (6.7)	191 (5.6)	0.05	202 (5.9)	190 (5.6)	0.02
Retinal detachments and breaks	535 (5.8)	154 (4.5)	0.06	156 (4.6)	152 (4.5)	0.01
Heart failure	971 (10.5)	269 (7.9)	0.09	256 (7.5)	269 (7.9)	0.01
Atrial fibrillation and flutter	510 (5.5)	156 (4.6)	0.05	136 (4.0)	156 (4.6)	0.03
Asthma	483 (5.2)	143 (4.2)	0.05	150 (4.4)	143 (4.2)	0.01
Other chronic obstructive pulmonary disease	351 (3.8)	108 (3.2)	0.04	100 (2.9)	108 (3.2)	0.01
Cerebral infarction	400 (4.3)	117 (3.4)	0.05	123 (3.6)	117 (3.4)	0.01
Medication, n (%)						
Bevacizumab	3578 (38.9)	898 (26.2)	0.27	892 (26.1)	897 (26.3)	0.01
Alpha blockers	588 (6.4)	198 (5.8)	0.03	205 (6.0)	198 (5.8)	0.01
Anti-diabetic medications	5416 (58.8)	1775 (51.9)	0.14	1682 (49.3)	1772 (51.9)	0.05
Antiplatelet medications	2239 (24.3)	809 (23.6)	0.02	751 (22.0)	800 (23.5)	0.03
Aspirin	2001 (21.7)	751 (21.9)	0.01	705 (20.7)	742 (21.8)	0.03
Beta blockers	3163 (34.4)	1086 (31.7)	0.06	1029 (30.2)	1077 (31.6)	0.03
Calcium channel blockers	2245 (24.4)	706 (20.6)	0.09	680 (19.9)	704 (20.6)	0.02
Diuretics	2853 (31.0)	1029 (30.1)	0.02	955 (28.0)	1022 (30.0)	0.04
HMG CoA reductase inhibitors	3842 (41.7)	1247 (36.4)	0.11	1192 (34.9)	1243 (36.4)	0.03
NSAIDs	1738 (18.9)	481 (14.1)	0.13	458 (13.4)	481 (14.1)	0.02
RAAS Inhibitors	3892 (42.3)	1362 (39.8)	0.05	1267 (37.1)	1355 (39.7)	0.05
Corticosteroids	2835 (30.8)	824 (24.1)	0.15	777 (22.8)	823 (24.1)	0.03
Laboratory data (mean ± SD)						
HbA1c	8.0 ± 1.9	8.1 ± 1.9	0.02	8.1 ± 2.0	8.1 ± 1.9	0.02
Creatinine [Mass/volume] in serum, plasma or blood	1.3 ± 2.7	1.4 ± 4.4	0.03	1.3 ± 0.8	1.4 ± 4.4	0.04
Blood pressure, systolic	137.7 ± 21.5	136.2 ± 20.6	0.07	136.3 ± 21.3	136.2 ± 20.6	0.01
BMI	31.5 ± 6.7	31.2 ± 6.8	0.04	31.4 ± 6.7	31.2 ± 6.8	0.02

*SMD* standardised mean difference, *PS* propensity score, *SD* standard deviation, *HMG CoA* 3-Hydroxy-3-methylglutaryl-CoA, *HbA1c* Glycated Haemoglobin, *BMI* body mass index.

### Subgroup and sensitivity analyses

To assess the significance of specific risk factors we conducted several subgroup analyses, including restriction of age to patients aged >40 years, sex, and history of CKD and hypertension. Focusing on patients with severe disease conditions, we analysed patients who received more than five injections to assess the impact of aflibercept on acute kidney injury or ESRD. All data management and statistical analyses were conducted on the TriNetX platform using SAS, version 9.4 (SAS Institute).

## Results

### Baseline characteristics

**Fig. 1:**
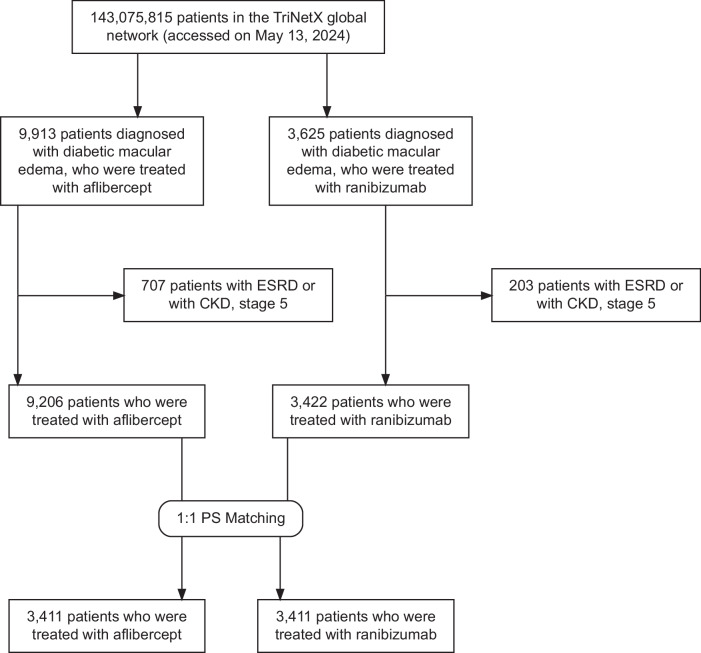
Cohort selection algorithm. *ESRD: end-stage renal disease, CKD chronic kidney disease, PS propensity score.

Initially, the raw study cohort comprised 9206 aflibercept users and 3422 ranibizumab users (Fig. 1). At baseline, there were no significant differences between the two groups with regard to age (63.7 ± 11.9 years vs. 64.0 ± 12.6 years) and proportion of males (50.8% vs. 51.6%).

After propensity score matching, the two matched groups had 3411 patients each, with the two groups very similar as regards mean age (aflibercept vs. ranibizumab: 63.9 ± 12.1 years vs. 64.0 ± 12.6 years) and proportion of males (51.4% vs. 51.6%). Similarly, there were no significant differences between the two groups as regards comorbidities, medication use, haemoglobin A1c levels (8.1 ± 2.0 vs. 8.1 ± 1.9), serum creatinine levels (1.3 ± 0.8 vs. 1.4 ± 4.4), systolic blood pressures (136.3 ± 21.3 mmHg vs. 136.2 ± 20.6 mmHg) and BMI (31.4 ± 6.7 vs. 31.2 ± 6.8) (Table 1).

### Primary analysis

The event rates for AKI were 25.3% and 34.9% in the aflibercept and ranibizumab groups, respectively, while for ESRD they were 10.7% and 15.2%, also respectively. The event rate for mortality in the aflibercept group was 17.0% (579 events among 3411 patients), while in the ranibizumab group it was 31.8% (1083 events among 3411 patients) (Table 2). Aflibercept was not associated with an increased risk of AKI (HR: 0.97, 95% CI: 0.89–1.06) or ESRD (HR: 0.95, 95% CI: 0.83–1.09), when compared to ranibizumab. In the matched cohorts, 579 patients (17.0%) receiving aflibercept and 1083 patients (31.8%) receiving ranibizumab died during follow-up. Thus, the respective crude and adjusted hazard ratios for mortality were 1.04 (95% CI: 0.95–1.13) and 0.99 (95% CI: 0.89–1.10) (Table 2). In the aflibercept and ranibizumab groups, respectively, the median time to the occurrence of AKI was 553 days and 949 days, while to the occurrence of ESRD it was 580 days and 982 days.

**Table 2:** Comparative risks of renal events.

	Aflibercept		Ranibizumab		HR (95% CI)	
	***N*** = **3411**		***N*** = **3411**			
**Outcomes**	Number of events	Event Rate* (%)	Number of events	Event Rate (%)	Crude	PS matching**
**ESRD**	364	10.7	517	15.2	1.12 (1.01-1.25)	0.95 (0.83-1.09)
**Acute kidney injury**	864	25.3	1192	34.9	1.17 (1.09-1.26)	0.97 (0.89-1.06)
**Mortality**	579	17.0	1083	31.8	1.04 (0.95-1.13)	0.99 (0.89-1.10)

*The discrepancy between lower event rates and hazard ratios was because the cox proportional hazards model accounts for follow-up time and person-time at risk, whereas the event rate does not.

** Adjusted by propensity score generated using the covariates listed in Table 1.

*ESRD* end-stage renal disease, *CI* confidence interval, *HR* hazard ratio, *PS* propensity score

### Subgroup analysis

**Fig. 2:**
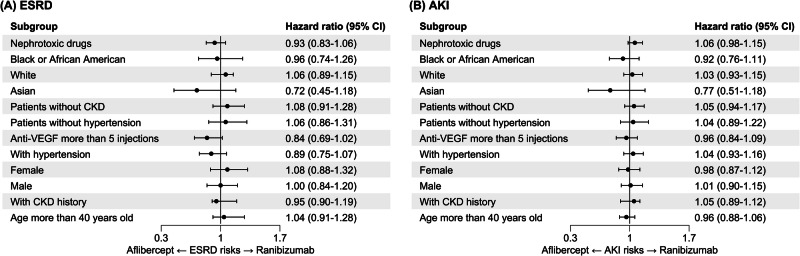
Subgroup analysis for (A) ESRD and (B) AKI. ESRD end-stage renal disease, CKD chronic kidney disease, VEGF vascular endothelial growth factor.

The subgroup analyses yielded results that were consistent with those of the primary analysis. Aflibercept showed no significant association with increased AKI risk in patients over 40 years (HR: 0.96, 95% CI: 0.88–1.06), in those with a history of CKD (HR: 1.05, 95% CI: 0.89–1.12), in males (HR: 1.01, 95% CI: 0.90–1.15), in patients with a hypertension history (HR: 1.04, 95% CI: 0.93–1.16), and in patients who received more than five injections (HR: 0.96, 95% CI: 0.84–1.09). For Asian patients, the hazard ratios (95% CIs) were as follows: AKI: 0.77 (0.51–1.18), ESRD: 0.72 (0.45–1.18), and mortality: 0.73 (0.41–1.28). For White patients, the hazard ratios (95% CIs) were: AKI: 1.03 (0.93–1.15), ESRD: 1.06 (0.89–1.15), and mortality: 1.14 (1.01-1.29), while for Black or African American patients, the hazard ratios (95% CIs) were: AKI: 0.92 (0.76–1.11), ESRD: 0.96 (0.74–1.26), and mortality: 1.19 (0.86–1.62). Details of the subgroup and sensitivity analyses are presented in Fig. 2.

### Sensitivity analysis

To address potential temporality issues between drug exposure and outcome occurrence, we performed a sensitivity analysis in which we applied a minimum 7-day latency window after treatment initiation. Under this restriction, the comparative risks between aflibercept and ranibizumab remained consistent with the primary analysis, with hazard ratios of 1.08 (95% CI, 0.94–1.24) for AKI, and 0.89 (95% CI, 0.76–1.04) for ESRD, indicating no material change in the observed associations. Additional sensitivity analyses stratified by ranibizumab dose (0.3 mg and 0.5 mg) and by injection number (≥3 and ≥6 injections) yielded hazard ratios consistently close to unity, with no clear dose- or exposure-dependent pattern observed for AKI, ESRD, or all-cause mortality (Supplementary Table 5). To further assess potential ethnic heterogeneity, we examined baseline characteristics within major ethnic subgroups before and after propensity score matching, and found good covariate balance within each stratum following matching (Supplementary Tables 6–8).

## Discussion

Using data from the global, federated TriNetX health research network we found no association between aflibercept use and an increased risk of AKI or ESRD in patients with DMO, when compared to ranibizumab use. Our primary findings, supported by consistent results from subgroup analyses, suggested that aflibercept and ranibizumab, used to treat patients with DMO, show similar risk profiles with regard to AKI and ESRD. The high event rates of AKI (25.3% and 34.9%) and ESRD (10.7% and 15.2%) observed in our propensity-matched cohorts are reflective of the severe underlying systemic microvascular disease in this patient population. DMO itself is a severe complication of diabetic retinopathy, which shares a common microvascular pathology with diabetic kidney disease (DKD). Recent real-world evidence has demonstrated that the presence of DMO is a significant independent predictor of renal failure, including a substantially increased risk of ESRD, dialysis, and renal transplantation in patients with diabetes and CKD [16, 17], which helps to explain the high incidence of these events in our study population.

Our findings remained consistent across multiple sensitivity and subgroup analyses. Systemic nephrotoxic medication classes that were reliably captured across sites, such as NSAIDs, ACEIs and ARBs, were incorporated into the propensity score model and matched between the treatment groups, ensuring balanced baseline renal risk. Pre-existing CKD status was likewise included as a covariate and further examined in stratified analyses, which showed similar trends and indicated that baseline renal impairment did not materially modify the treatment effect. Because more detailed information on other nephrotoxic agents, including specific antibiotics and antivirals, was only inconsistently available across data partners, their potential influence was assessed in supplementary subgroup analyses rather than within the primary matching procedure. The results across these additional subgroups remained robust, reinforcing the stability of our main conclusions. We recognize that treatment selection in real-world practice may also be influenced by socioeconomic factors, healthcare access, and evolving market adoption patterns—particularly in multinational settings; however, extensive propensity score matching incorporating comorbidity burden, medication use, and laboratory parameters proxies was applied to mitigate confounding by baseline health status. In view of the observed high event rate of adverse renal events, close monitoring of the renal function of patients receiving anti-VEGF treatment is crucial to reduce the risk of adverse renal outcomes among patients with DMO.

One randomized controlled trial, the landmark DRCR.net Protocol T study, directly compared aflibercept, ranibizumab and bevacizumab, and found all three to significantly improve visual acuity, with aflibercept more favourable for patients with worse baseline acuity [18]. However, evidence regarding adverse renal effects from aflibercept and ranibizumab remains limited [19, 20]. Initially, our real-world analysis found increased risks of AKI, ESRD and mortality with aflibercept, when compared to ranibizumab. After balancing the baseline characteristics of the two groups through PS matching, however, these elevated risks were no longer in evidence. This suggested that the increased incidence of adverse renal events that is subjectively observed by physicians in patients using aflibercept may, in fact, be a result of unbalanced baseline demographics.

Patients receiving aflibercept or ranibizumab may differ significantly with regard to characteristics like sex, age, underlying health conditions and other comorbidities, which would likely confound any investigation. Existing literature points to a higher incidence of renal impairment among males, which we initially observed in our crude hazard ratios as a sex-based discrepancy. Similarly, the prevalence of hypertension may have been another confounding factor in our study population. Systemic conditions such as stroke, ischemic heart disease, and retinal occlusion are associated with hypertension, as is renal impairment, and hence the prevalence of hypertension may also have influenced our crude comparative results.

The fact that the increase in AKI risk was no longer observable after we applied PS matching to control for differences at baseline clearly illustrates how an observed association does not indicate true causation, and underscores the need to take such baseline differences into account in observational studies. The results from our subgroup analyses that were consistent with our primary analyses further confirmed that risks of adverse renal effects were indeed similar for both medications, since they implied that medication could be selected to address the needs, preferences and clinical circumstances of each individual patient [21]. Furthermore, while our study results suggest that none of the anti-VEGF agents studied pose increased risks of adverse renal effects compared to others, the observed overall high incidence of adverse renal events emphasises the need to closely monitor renal function during treatment. If renal function is found to be in unusually rapid decline, the possibility of a relationship with anti-VEGF therapy should not be ignored. Vigilance toward adverse renal events is crucial in ensuring patient safety, regardless of which medication is used.

Although exportable summaries of calendar-year distribution data for treatment initiation were not available, relative timing information anchored to treatment initiation dates enabled valid time-to-event and latency-based analyses. Our propensity score model incorporated strong proxies for disease severity and chronicity—such as microvascular and macrovascular complications (e.g., neuropathy, peripheral vascular disease) and insulin use, which often reflects long-standing or advanced diabetes. These variables helped ensure that the underlying diabetes burden was well balanced between the groups. We also recognize that patterns of drug adoption may vary by region, and that exact calendar-year data are not uniformly accessible within the de-identified TriNetX network, limiting our ability to adjust directly for treatment year. We acknowledge that the lack of calendar year information in TriNetX may limit the ability to evaluate potential secular trends in AKI reporting rates. Although recent pharmacovigilance analyses of spontaneous reporting systems have suggested temporal declines in AKI reports [22], this limitation is unlikely to materially affect the comparative risk estimates between aflibercept and ranibizumab, as both agents would be subject to similar background reporting dynamics over time. To address potential temporal confounding, we conducted an additional sensitivity analysis restricted to the period 2020–2023, during which both agents were well-established in routine practice. The results were consistent with the primary analysis, suggesting that our findings were unlikely to be mere reflections of the differences in clinical era (AKI - HR: 1.23, 95% CI: 0.94–1.61; ESRD - HR: 0.91, 95% CI: 0.64–1.30). Certain parameters that may influence renal outcomes, such as baseline visual acuity, diabetes duration, and the exact age at treatment initiation, were not available in standardised form within the TriNetX Global Collaborative Network. Likewise, detailed information on systemic nephrotoxic medication use was inconsistently captured across data partners. The analytic framework therefore focused on a broad set of available covariates with established renal relevance, including demographic factors, comorbidities and laboratory results (e.g., HbA1c, serum creatinine, BMI and systolic blood pressure). Pre-existing ESRD and CKD stage 5 were excluded from cohort entry, and propensity score matching yielded excellent balance across all measurable characteristics. Taken together, these measures minimised residual confounding and supported a robust comparison of the risks of AKI and ESRD between aflibercept and ranibizumab.

The study utilized data from the TriNetX Global Collaborative Network, linking healthcare organisations from multiple regions worldwide. Consequently, the population analysed was not confined to Taiwan but represented a multi-national, ethnically-mixed cohort. Although ethnicity-specific data were not uniformly available across all sites, the inclusion of diverse healthcare systems enhanced the external validity of our findings. Nevertheless, subtle regional or pharmacogenomic variations cannot be entirely excluded and should be considered when extrapolating these results to specific populations. We included all-cause mortality as a secondary exploratory endpoint to provide a comprehensive overview of systemic safety, rather than to imply a direct causal association with intravitreal anti-VEGF therapy. Within the TriNetX Global Collaborative Network, mortality events are recorded as de-identified all-cause outcomes without specification of aetiology or timing relative to injection. The comparable mortality rates between aflibercept and ranibizumab users therefore reflect the general health profile of patients with diabetic macular oedema, rather than treatment-related fatalities. These findings reinforce the systemic safety of both agents, while highlighting the need for ongoing multidisciplinary management of this comorbidity-burdened population.

Rather than conducting separate comparisons of baseline characteristics across the ethnic groups, we evaluated risk estimates by ethnicity through stratified analyses, with covariate balance ensured within each matched comparison, consistent with the study’s comparative safety objective. Our findings differ from our earlier study [23] in a homogeneous East-Asian population and highlight the potential influence of ancestry on renal safety profiles associated with intravitreal anti-VEGF therapy. In leveraging the multi-national, multi-ethnic TriNetX Global Collaborative Network, which includes patients of Asian, White and Black or African-American descent, this analysis reveals how racial and genetic diversity may contribute to variations in renal outcomes. These findings suggest that population-specific factors, rather than universal drug effects, may partly explain discrepancies across studies.

Influenced by environmental, biological and genetic factors, different ethnicities are susceptible to medications in different ways. Drug metabolism can be affected by genetic polymorphisms that determine how effectively a drug is processed, and hence any risks of adverse effects [24–28]. Across different ethnic groups, some of the more recently introduced anti-VEGF therapies to treat maculopathy have displayed differing risk profiles for adverse events. For example, brolucizumab, introduced in 2020 as a new anti-VEGF agent to treat wet age-related macular degeneration and DMO, has been associated with a higher incidence of retinal vasculitis and inflammatory reactions in Asian populations, compared to other groups [29]. Furthermore, in Western countries, high-dose aflibercept has yielded effective and safe outcomes, but in Japan it has recently been associated with severe cases of vasculitis [30]. These findings underscore the need to take ethnic differences into account when evaluating anti-VEGF treatments and their safety profiles. While the majority of our study population from the TriNetX database was White, previous research was focused primarily on an Asian population [31]. East Asian genetic profiles appear to have fewer variants associated with increased kidney disease susceptibility, compared to Caucasians [32], and this may affect the risk profiles of the anti-VEGF treatments, producing variations in medication response as a result of ethnic and demographic factors.

While the large sample size provided to our study by the TriNetX platform enabled robust statistical power and enhanced the generalisability of our findings across diverse patient populations, some limitations of this study remain. The full capture by the TriNetX platform of all relevant clinical nuances, such as details on healthcare settings or treatment adherence might not be complete. Also, TriNetX incorporates structured laboratory and physiological data, including HbA1c, serum creatinine, BMI and blood pressure, which were unavailable in the claims-based NHIRD in our previous study [23]. The wide standard deviations observed for certain baseline laboratory variables, such as serum creatinine, reflect real-world heterogeneity across the multiple healthcare organisations within the TriNetX network, rather than data entry errors. These variables enabled adjustment for key metabolic and renal risk factors, and thereby improved our control of confounding and the internal validity of our comparative analyses. We recognize the value of comparing our event rates with an anti-VEGF-naïve diabetic cohort to better contextualise absolute risk. We also acknowledge that reliance on ICD-coded AKI in EMR data may lead to the inclusion of mild or transient creatinine elevations that meet biochemical definitions but do not necessarily reflect clinically severe events typically observed in trials. Importantly, any such over-capture would occur non-differentially across treatment groups, preserving the validity of the relative risk comparison. Overall, our findings align with prior large real-world studies demonstrating no meaningful differences in renal outcomes—including no increased risk of AKI or ESRD—between anti-VEGF agents in patients with diabetic macular oedema or proliferative diabetic retinopathy. As the primary aim of this study was to compare the safety of aflibercept and ranibizumab, the application of rigorous propensity score matching effectively minimised baseline imbalances and confirmed their broadly comparable renal safety profiles. To summarize, by ensuring comparable baseline characteristics between the treatment groups through target trial emulation and propensity score matching we effectively reduced confounding, and thereby strengthened the validity of our comparative analysis [33].

## Conclusion

After adjustment for measurable confounders, this study found no significant difference in the risks of AKI, ESRD or mortality between patients treated with aflibercept or ranibizumab for diabetic macular oedema. Future clinical trials are warranted to validate these findings.

Supplemental material is available at Eye’s website.

## Summary

### What was known before

- There was only limited evidence regarding the comparative risks of adverse renal events in patients with diabetic macular oedema (DMO) receiving either aflibercept or ranibizumab.

### What this study adds

- After adjusting for confounders, this study found no significant difference in the risks of acute kidney injury (AKI), end-stage renal disease (ESRD) and mortality between patients treated with aflibercept and those treated with ranibizumab. Therefore, in DMO patients, both aflibercept and ranibizumab have comparable risk profiles as regards AKI and ESRD.

## Supplementary information

Supplementary Material

## Data Availability

The datasets generated during and/or analysed during the current study are available upon reasonable request from TriNetX.
