# Supplementary material for: Comparison of adverse renal events between ranibizumab and aflibercept in patients with diabetic macular oedema: A global network study
Source: Eye (Lond). 2026 Apr 22;40(10):1446–53. doi: 10.1038/s41433-026-04459-4 (PMC13342618; doi:10.1038/s41433-026-04459-4)
Supplement: Supplementary file 1 — Supplementary Material [file 41433_2026_4459_MOESM1_ESM.docx]

**Supplementary Material**

Lee WA, Tsai DHT, Cheng MCY, et al. Comparison of Adverse Renal Events Between Ranibizumab and Aflibercept in Patients with Diabetic Macular Edema: A global network study

**Supplementary Notes**

| **Note 1** | [Overview of the TriNetX network.](#Sup_N1) | P.2 |
| --- | --- | --- |
| **Note 2** | [Rationale for reporting standardized mean differences.](#Sup_N2) | P.2 |

1. **Supplementary Figures**

| **Figure 1** | [Differences in cumulative incidence of (A) acute kidney injury (AKI), (B) end-stage renal disease (ESRD), and (C) mortality, between the ranibizumab and aflibercept groups, before propensity score matching.](#Sup_F1) | P.3 |
| --- | --- | --- |

1. **Supplementary Tables**

| **Table 1** | [Specification of a target trial emulation comparing the hazards of kidney complications as a result of treatment with aflibercept vs. ranibizumab, using real-world data from TriNetX.](#Sup_T1) | P. 4 |
| --- | --- | --- |
| **Table 2** | [ICD-10 diagnostic codes for diseases.](#Sup_T2) | P. 5 |
| **Table 3** | [ATC Codes for medications.](#Sup_T3) | P. 6 |
| **Table 4** | [ICD-10 diagnostic codes for main outcomes.](#Sup_T4) | P. 6 |
| **Table 5** | [Sensitivity analyses by ranibizumab dose and number of injections.](#Sup_T5) | P. 7 |
| **Table 6-8** | [Baseline characteristics of different ethnic patients before and after propensity score matching.](#Sup_T6) | P. 8-12 |

1. **References** (P. 13)

**Supplementary Note 1:** Overview of the TriNetX network.

Dedicated to improving patient outcomes, TriNetX serves as a global nexus for health research by aggregating and harmonizing electronic medical records (EMR) from international healthcare institutions. Built on a rapidly expanding network of hospitals, research centers, and industry partners, the platform integrates structured and unstructured real-world data—including diagnoses, procedures, medications, laboratory values, and outcomes—into a unified analytic framework. This vast, heterogeneous, and continuously updated repository of longitudinal clinical data supports a wide spectrum of research activities, ranging from cohort discovery and feasibility assessments to pharmacoepidemiologic inquiries, comparative effectiveness studies, and safety surveillance.

Through sophisticated data normalization pipelines, ontology mapping, and quality-control measures, TriNetX minimizes the variability inherent in multi-institutional datasets, allowing researchers to perform reproducible analyses with confidence. Its federated network architecture enables computation to occur behind each institution’s firewall, ensuring that patient-level data never leave the host site. As a result, the system provides both analytical flexibility and stringent compliance with international data-protection standards.

Importantly, TriNetX maintains a secure, de-identified environment that safeguards privacy while still allowing investigators to capture real-world clinical complexity across diverse populations and settings. This infrastructure fosters meaningful collaboration among academic investigators, healthcare providers, and life-science organizations, empowering them to generate timely, policy-relevant evidence. By helping to transform disparate clinical information into actionable insights, TriNetX fundamentally enhances the capacity of the global research community to address unmet clinical needs and accelerate innovations that improve patient care.

Patients included in this study initiated their treatment with aflibercept or ranibizumab during routine clinical care after regulatory approval of both agents in their respective regions. Although exact calendar-year distribution data for treatment initiation are not provided as structured exportable variables within TriNetX, the study population predominantly reflects contemporary post-approval practice. To further address potential temporal confounding, we conducted a sensitivity analysis restricted to the period 2020–2023, which yielded results consistent with the primary analysis.

**Supplementary Note 2:** Rationale for reporting standardized mean differences.

To evaluate baseline covariate balance between the treatment groups, we relied on the standardized mean difference (SMD) rather than p-values. This approach aligns with established best practices for propensity score analysis, as the SMD quantifies the magnitude of difference independently of sample size, whereas significance testing is sensitive to statistical power and may yield significant results for negligible differences in large samples. [1-5]

**Supplementary Figure 1.**

1. Survival curve, comparing acute kidney injury in the ranibizumab and aflibercept groups.


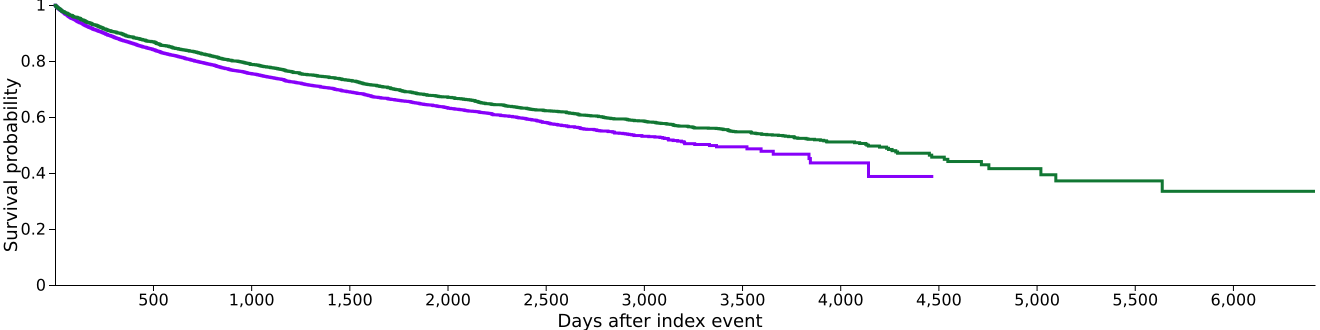


1. Survival curve, comparing ESRD in the ranibizumab and aflibercept groups.


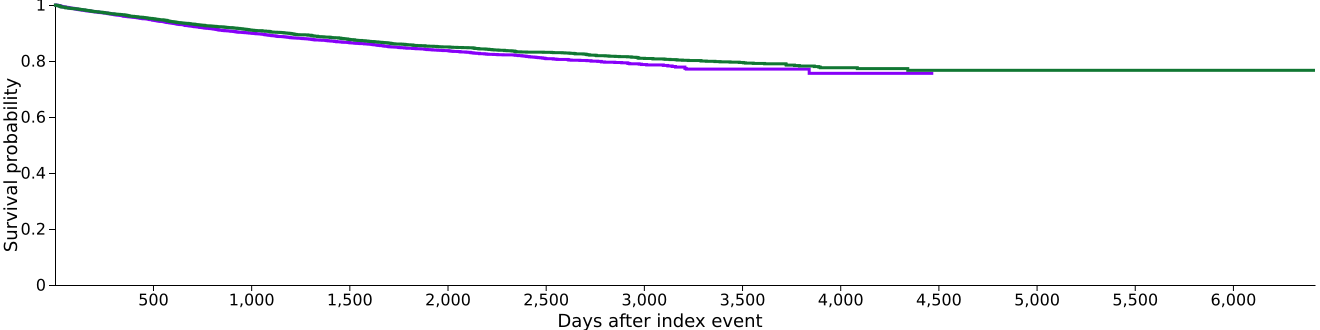


(C) Survival curve, comparing mortality in the ranibizumab and aflibercept groups.


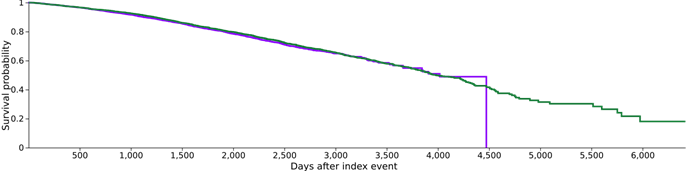


**Supplementary Table 1.** Specification of a target trial emulation comparing the hazards of kidney complications as a result of treatment with aflibercept vs. ranibizumab, using real-world data from TriNetX.

| **Component** | **Target trial** | **Emulated trial using real-world data** |
| --- | --- | --- |
| Aim | Compare risks of adverse renal effects, such as acute kidney injury and end-stage renal disease in patients with diabetic macular edema. | Same |
| Eligibility | Adult patients with diagnosed diabetic macular edema, aged 20 yrs or above, with no history of chronic kidney disease or end stage renal disease. | Same |
| Treatment strategies | 1. Administer ranibizumab  2. Administer aflibercept | Same |
| Treatment assignment | Patients randomly assigned to either treatment group (equal probability of assignment to either treatment group). | Use propensity score method to mimic randomization of assignment to either treatment group. |
| Follow-up | Follow-up from treatment assignment until occurrence of diabetes complications, death, loss to follow-up or end of the study period, whichever occurred first. | Same |
| Outcome | Acute kidney injury, dialysis or renal transplant, death from renal causes, hospitalization or ER visits due to renal issues. | Same |
| Causal contrast | Intention-to-treat effect, indicating the effect of being assigned to either aflibercept or ranibizumab at baseline, regardless of whether patients maintained the assigned treatment thereafter. | Same (using as-started analysis, which is an analog of ITT) |
| Statistical analysis | Cox proportional hazards model | Same |

**Abbreviations: ITT: intention-to-treat**

**Supplementary Table 2.** ICD10 codes for each disease.

| Disease Code | ICD-10 | Comments |
| --- | --- | --- |
| Diabetic macular edema | E11.311 | Main Disease |
|  |  |  |
| Acute myocardial infarction | I21 | 365 days before Index date |
| Asthma | J45 |  |
| Atrial fibrillation and flutter | I48 |  |
| Cataract | H25-H26 |  |
| Coagulopathy | D68 |  |
| Chronic obstructive pulmonary disease | J44 |  |
| Diabetes mellitus | E10, E11 |  |
| Dyslipidemia | E78 |  |
| Glaucoma | H40-H42 |  |
| Heart failure | I50 |  |
| Hypertension | I10 |  |
| Ischemic heart disease | I20-I25 |  |
| Ischemic stroke | I63 |  |
| Liver diseases | K70-K77 |  |
| Influenza and pneumonia | J09-J19 |  |
| Other disorders of kidney and ureter | N25-N29 |  |
| Chronic kidney disease, stage 5 | N18.5 | Exclusion criteria |
| End stage renal disease | N18.6, Z99.2 |  |
| Acute kidney injury | N28.9, N17, N19 |  |

**Supplementary Table 3.** ATC codes for medications.

| Medications | TriNetX codes | Comments |
| --- | --- | --- |
| Ranibizumab | **RXNORM:595060** | Main drugs |
| Aflibercept | **RXNORM:1232150** |  |
| Alpha-blockers | CV150 | 365 days before Index date |
| Anti-diabetic medications | HS500 |  |
| Antiplatelet medications | B01AC |  |
| Anti-thyroid agents, thyroxin | H03A, H03BA, H03BB |  |
| Aspirin | 1191 |  |
| Beta-blockers | C07A |  |
| Calcium channel blockers | C08 |  |
| Digoxin | C01AA05 |  |
| Diuretics | C03 |  |
| HMG-CoA reductase inhibitors | C10AA |  |
| Hormone | G03FA, G03FB, G03C, G03D |  |
| NSAIDs | S01BC |  |
| RAAS Inhibitors | C09 |  |
| Corticosteroids | S01BA |  |
| Abbreviations: NSAIDs: Non-steroidal anti-inflammatory drugs; HMG-CoA: 3-hydroxy-3-methylglutaryl-coenzyme A reductase; RAAS: renin-angiotensin-aldosterone system | | |

**Supplementary Table 4.** ICD-10 diagnostic codes for main outcomes.

| **Outcome** | **ICD-10** |
| --- | --- |
| Acute kidney injury | N28.9, N17, N19 |
| End stage renal disease | N18.6, Z99.2 |

**Supplementary Table 5.** Sensitivity analyses by ranibizumab dose and number of injections.

Comparative risks of renal outcomes and mortality associated with aflibercept vs. ranibizumab, stratified by ranibizumab dose and number of injections.

| **Stratification** | **ESRD**  **HR (95% CI)** | **AKI**  **HR (95% CI)** | **All-cause Mortality**  **HR (95% CI)** |
| --- | --- | --- | --- |
| **Ranibizumab 0.3 mg** | 0.88 (0.71–1.09) | 0.94 (0.81–1.10) | 0.88 (0.68–1.14) |
| **Ranibizumab 0.5 mg** | 0.96 (0.76–1.22) | 0.92 (0.78–1.07) | 0.96 (0.80–1.16) |
| **≥3 injections** | 0.89 (0.76–1.04) | 1.01 (0.91–1.13) | 1.00 (0.87–1.14) |
| **≥6 injections** | 0.86 (0.71–1.05) | 0.88 (0.77–1.01) | 0.85 (0.71–1.02) |

**Supplementary Table 6.** Baseline characteristics of White patients before and after propensity score matching.

| **White** | **Before PS matching** | | | **After PS matching** | | |
| --- | --- | --- | --- | --- | --- | --- |
| **Variables** | **Aflibercept** | **Ranibizumab** | **SMD** | **Aflibercept** | **Ranibizumab** | **SMD** |
| Male sex | 55.3% | 52.1% | 0.06 | 52.1% | 52.1% | 0 |
| Essential hypertension | 55.1% | 48.0% | 0.14 | 45.1% | 48.1% | 0.06 |
| Disorders of lipoprotein metabolism and other lipidemias | 46.6% | 38.5% | 0.16 | 36.6% | 38.6% | 0.04 |
| Age-related cataract | 36.1% | 27.9% | 0.18 | 28.7% | 27.9% | 0.02 |
| Acute kidney failure and chronic kidney disease | 21.8% | 16.1% | 0.15 | 16.5% | 16.1% | 0.01 |
| Ischemic heart diseases | 20.3% | 16.7% | 0.09 | 15.5% | 16.7% | 0.03 |
| Glaucoma | 17.1% | 14.3% | 0.08 | 13.7% | 14.4% | 0.02 |
| Heart failure | 10.9% | 7.6% | 0.11 | 6.9% | 7.6% | 0.03 |
| Other disorders of kidney and ureter | 6.7% | 5.9% | 0.04 | 5.9% | 5.9% | 0 |
| Atrial fibrillation and flutter | 7.3% | 5.5% | 0.07 | 5.4% | 5.5% | 0.01 |
| Retinal detachments and breaks | 5.8% | 4.8% | 0.04 | 5.2% | 4.8% | 0.02 |
| Cerebral infarction | 4.1% | 3.7% | 0.02 | 4.1% | 3.6% | 0.02 |
| Asthma | 5.0% | 3.9% | 0.05 | 3.5% | 3.9% | 0.02 |
| Influenza and pneumonia | 3.2% | 2.7% | 0.03 | 2.7% | 2.7% | 0 |
| Other chronic obstructive pulmonary disease | 4.5% | 3.2% | 0.07 | 2.5% | 3.2% | 0.04 |
| Diseases of liver | 4.4% | 2.5% | 0.10 | 2.4% | 2.5% | 0.01 |
| Acute myocardial infarction | 3.3% | 2.3% | 0.06 | 2.2% | 2.3% | 0 |
| **Medication (%)** | | | | | | |
| Blood glucose regulation agents | 59.9% | 49.5% | 0.21 | 47.5% | 49.6% | 0.04 |
| Agents acting on the renin-angiotensin system | 42.3% | 37.2% | 0.10 | 35.4% | 37.2% | 0.04 |
| HMG CoA reductase inhibitors | 42.1% | 35.4% | 0.14 | 33.4% | 35.4% | 0.04 |
| Bevacizumab | 42.3% | 31.0% | 0.24 | 31.6% | 31.1% | 0.01 |
| Beta blocking agents | 33.4% | 30.9% | 0.05 | 28.8% | 30.8% | 0.04 |
| Diuretics | 29.7% | 28.7% | 0.02 | 27.3% | 28.5% | 0.03 |
| Corticosteroids, plain | 31.5% | 24.9% | 0.15 | 25.5% | 25.0% | 0.01 |
| Platelet aggregation inhibitors excl. heparin | 23.0% | 21.9% | 0.03 | 20.1% | 21.8% | 0.04 |
| Aspirin | 20.5% | 19.7% | 0.02 | 18.5% | 19.6% | 0.03 |
| Calcium channel blockers | 21.5% | 17.6% | 0.10 | 16.5% | 17.6% | 0.03 |
| Anti-inflammatory agents, non-steroids | 17.7% | 13.9% | 0.11 | 13.3% | 13.9% | 0.02 |
| Alpha blockers | 6.6% | 5.6% | 0.05 | 4.9% | 5.6% | 0.03 |
| **Laboratory data (mean ± SD)** | | | | | | |
| Blood Pressure, Systolic | 135.3 ± 20.8 | 136.0 ± 20.9 | 0.04 | 135.2 ± 20.4 | 136.0 ± 20.9 | 0.04 |
| BMI | 32.9 ± 7.6 | 32.5 ± 7.3 | 0.05 | 33.0 ± 7.9 | 32.5 ± 7.3 | 0.06 |
| Creatinine | 1.4 ± 5.9 | 1.4 ± 5.2 | 0.01 | 1.5 ± 8.0 | 1.4 ± 5.2 | 0.03 |
| HbA1c | 7.8 ± 1.7 | 8.0 ± 1.9 | 0.09 | 8.0 ± 1.9 | 8.0 ± 1.9 | 0.02 |

**Supplementary Table 7**. Baseline characteristics of Black or African American patients before and after propensity score matching.

| **Black or African American** | **Before PS matching** | | | **After PS matching** | | |
| --- | --- | --- | --- | --- | --- | --- |
| **Variables** | **Aflibercept** | **Ranibizumab** | **SMD** | **Aflibercept** | **Ranibizumab** | **SMD** |
| Male sex | 38.8% | 40.2% | 0.03 | 41.0% | 40.3% | 0.01 |
| Essential hypertension | 66.4% | 64.4% | 0.04 | 64.2% | 64.4% | 0 |
| Disorders of lipoprotein metabolism and other lipidemias | 48.1% | 46.1% | 0.04 | 48.7% | 46.2% | 0.05 |
| Age-related cataract | 45.7% | 34.3% | 0.23 | 36.0% | 34.4% | 0.03 |
| Glaucoma | 29.9% | 26.9% | 0.07 | 26.7% | 26.9% | 0 |
| Acute kidney failure and chronic kidney disease | 26.9% | 23.7% | 0.08 | 24.4% | 23.7% | 0.02 |
| Ischemic heart diseases | 15.3% | 14.1% | 0.04 | 13.4% | 14.1% | 0.02 |
| Heart failure | 13.9% | 10.7% | 0.1 | 11.1% | 10.7% | 0.01 |
| Asthma | 7.6% | 7.5% | 0 | 8.4% | 7.3% | 0.04 |
| Other disorders of kidney and ureter | 8.4% | 6.4% | 0.07 | 7.7% | 6.4% | 0.05 |
| Retinal detachments and breaks | 7.0% | 5.3% | 0.07 | 4.8% | 5.4% | 0.02 |
| Cerebral infarction | 6.2% | 4.5% | 0.08 | 4.5% | 4.5% | 0 |
| Atrial fibrillation and flutter | 4.9% | 3.6% | 0.07 | 3.2% | 3.6% | 0.02 |
| Other chronic obstructive pulmonary disease | 4.1% | 3.0% | 0.06 | 3.2% | 3.0% | 0.01 |
| Acute myocardial infarction | 2.3% | 2.9% | 0.04 | 2.7% | 2.9% | 0.01 |
| Influenza and pneumonia | 3.3% | 2.3% | 0.06 | 2.1% | 2.3% | 0.01 |
| Diseases of liver | 2.7% | 2.0% | 0.05 | 1.8% | 2.0% | 0.01 |
| **Medication (%)** | | | | | | |
| Blood glucose regulation agents | 65.3% | 64.2% | 0.21 | 63.1% | 64.2% | 0.04 |
| Agents acting on the renin-angiotensin system | 49.1% | 52.9% | 0.10 | 52.9% | 52.8% | 0.04 |
| HMG CoA reductase inhibitors | 48.3% | 47.2% | 0.14 | 46.2% | 47.2% | 0.04 |
| Bevacizumab | 42.1% | 41.8% | 0.24 | 36.7% | 41.7% | 0.01 |
| Beta blocking agents | 42.5% | 39.5% | 0.05 | 35.5% | 39.6% | 0.04 |
| Diuretics | 33.8% | 34.9% | 0.02 | 30.0% | 34.9% | 0.03 |
| Corticosteroids, plain | 28.8% | 27.8% | 0.15 | 28.5% | 27.8% | 0.01 |
| Platelet aggregation inhibitors excl. heparin | 25.7% | 25.3% | 0.03 | 25.7% | 25.3% | 0.04 |
| Aspirin | 33.2% | 24.7% | 0.02 | 25.5% | 24.8% | 0.03 |
| Calcium channel blockers | 36.8% | 20.5% | 0.10 | 19.3% | 20.5% | 0.03 |
| Anti-inflammatory agents, non-steroids | 22.8% | 17.8% | 0.11 | 17.7% | 17.8% | 0.02 |
| Alpha blockers | 6.0% | 5.0% | 0.05 | 3.0% | 5.0% | 0.03 |
| **Laboratory data (mean ± SD)** | | | | | | |
| Blood Pressure, Systolic | 141.6 ± 21.5 | 139.3 ± 20.1 | 0.11 | 141.3 ± 23.5 | 139.3 ± 20.1 | 0.09 |
| BMI | 32.6 ± 7.4 | 32.7 ± 8.1 | 0.01 | 31.6 ± 7.8 | 32.7 ± 8.1 | 0.14 |
| Creatinine | 1.5 ± 4.6 | 1.3 ± 0.6 | 0.07 | 1.3 ± 0.8 | 1.3 ± 0.6 | 0.08 |
| HbA1c | 8.4 ± 2.2 | 8.6 ± 2.2 | 0.09 | 8.4 ± 2.2 | 8.6 ± 2.2 | 0.09 |

**Supplementary Table 8**. Baseline characteristics of Asian patients before and after propensity score matching.

| **Asian** | **Before PS matching** | | | **After PS matching** | | |
| --- | --- | --- | --- | --- | --- | --- |
| **Variables** | **Aflibercept** | **Ranibizumab** | **SMD** | **Aflibercept** | **Ranibizumab** | **SMD** |
| Male sex | 54.6% | 57.5% | 0.06 | 60.2% | 56.3% | 0.08 |
| Essential (primary) hypertension | 45.4% | 38.7% | 0.13 | 38.8% | 38.8% | 0 |
| Disorders of lipoprotein metabolism and other lipidemias | 41.8% | 36.5% | 0.11 | 32.5% | 36.4% | 0.08 |
| Age-related cataract | 30.5% | 15.0% | 0.38 | 18.4% | 17.0% | 0.04 |
| Glaucoma | 18.4% | 12.4% | 0.17 | 16.0% | 15.0% | 0.03 |
| Acute kidney failure and chronic kidney disease | 18.4% | 21.1% | 0.07 | 16.0% | 18.4% | 0.06 |
| Ischemic heart diseases | 16.2% | 10.2% | 0.18 | 10.7% | 11.2% | 0.02 |
| Diseases of liver | 4.4% | 3.8% | 0.03 | 4.9% | 4.9% | 0 |
| Acute myocardial infarction | 2.7% | 3.8% | 0.06 | 4.9% | 4.9% | 0 |
| Influenza and pneumonia | 3.5% | 3.8% | 0.01 | 4.9% | 4.9% | 0 |
| Other disorders of kidney and ureter | 4.6% | 3.8% | 0.04 | 4.9% | 4.9% | 0 |
| Retinal detachments and breaks | 3.1% | 4.1% | 0.06 | 4.9% | 4.9% | 0 |
| Heart failure | 7.1% | 4.5% | 0.11 | 4.9% | 5.3% | 0.02 |
| Atrial fibrillation and flutter | 2.4% | 3.8% | 0.08 | 4.9% | 4.9% | 0 |
| Asthma | 2.2% | 3.8% | 0.09 | 4.9% | 4.9% | 0 |
| Other chronic obstructive pulmonary disease | 2.2% | 3.8% | 0.09 | 4.9% | 4.9% | 0 |
| Cerebral infarction | 4.2% | 4.9% | 0.03 | 4.9% | 4.9% | 0 |
| **Medication (%)** | | | | | | |
| Blood glucose regulation agents | 61.1% | 55.3% | 0.12 | 51.0% | 53.9% | 0.06 |
| Corticosteroids, plain | 35.8% | 57.1% | 0.44 | 47.6% | 48.1% | 0.01 |
| HMG CoA reductase inhibitors | 49.1% | 36.5% | 0.26 | 37.4% | 38.8% | 0.03 |
| Agents acting on the renin-angiotensin system | 40.5% | 32.7% | 0.16 | 33.0% | 31.6% | 0.03 |
| Calcium channel blockers | 27.7% | 28.6% | 0.02 | 26.7% | 26.7% | 0 |
| Platelet aggregation inhibitors excl. Heparin | 26.8% | 22.2% | 0.11 | 23.8% | 21.8% | 0.05 |
| Beta blocking agents | 34.3% | 24.1% | 0.23 | 23.8% | 24.8% | 0.02 |
| Aspirin | 22.6% | 19.9% | 0.06 | 21.4% | 19.4% | 0.05 |
| Diuretics | 24.6% | 22.6% | 0.05 | 19.4% | 20.9% | 0.04 |
| Anti-inflammatory agents, non-steroids | 15.9% | 12.8% | 0.09 | 12.6% | 13.1% | 0.01 |
| Alpha blockers/related | 7.7% | 11.7% | 0.13 | 9.2% | 7.8% | 0.05 |
| Bevacizumab | 24.8% | 6.8% | 0.51 | 8.7% | 8.7% | 0 |
| **Laboratory data (mean ± SD)** | | | | | | |
| Blood Pressure, Systolic | 132.6 ± 19.2 | 140.3 ± 21.9 | 0.37 | 134.7 ± 19.3 | 138.9 ± 21.8 | 0.20 |
| BMI | 26.0 ± 4.3 | 26.2 ± 4.7 | 0.04 | 26.3 ± 4.0 | 26.2 ± 4.8 | 0.03 |
| Creatinine | 1.3 ± 0.9 | 2.1 ± 5.9 | 0.20 | 1.4± 1.2 | 2.1 ± 6.8 | 0.16 |
| HbA1c | 7.9 ± 1.6 | 7.7 ± 1.5 | 0.15 | 7.9 ± 1.7 | 7.9 ± 1.6 | 0.01 |

SMD: standardized mean difference, PS: propensity score, SD: standard deviation, HMG CoA: 3-Hydroxy-3-methylglutaryl-CoA, HbA1c: Glycated Hemoglobin, BMI: body mass index.

**References:**

1. Austin PC, Grootendorst P, Anderson GM: **A comparison of the ability of different propensity score models to balance measured variables between treated and untreated subjects: a Monte Carlo study**. *Statistics in medicine* 2007, **26**(4):734-753.

2. Austin PC: **Type I error rates, coverage of confidence intervals, and variance estimation in propensity-score matched analyses**. *Int J Biostat* 2009, **5**(1):13-13.

3. Murad MH, Wang Z, Chu H, Lin L: **When continuous outcomes are measured using different scales: guide for meta-analysis and interpretation**. *Bmj* 2019, **364**:k4817.

4. Pustejovsky JE, Rodgers MA: **Testing for funnel plot asymmetry of standardized mean differences**. *Res Synth Methods* 2019, **10**(1):57-71.

5. Andrade C: **Mean Difference, Standardized Mean Difference (SMD), and Their Use in Meta-Analysis: As Simple as It Gets**. *The Journal of clinical psychiatry* 2020, **81**(5).
